# A Novel Computational Framework to Predict Disease-Related Copy Number Variations by Integrating Multiple Data Sources

**DOI:** 10.3389/fgene.2021.696956

**Published:** 2021-06-29

**Authors:** Lin Yuan, Tao Sun, Jing Zhao, Zhen Shen

**Affiliations:** ^1^School of Computer Science and Technology, Qilu University of Technology (Shandong Academy of Sciences), Jinan, China; ^2^School of Computer and Software, Nanyang Institute of Technology, Nanyang, China

**Keywords:** CNV, multi-omics data, path association analysis, stability selection, prostate cancer

## Abstract

Copy number variation (CNV) may contribute to the development of complex diseases. However, due to the complex mechanism of path association and the lack of sufficient samples, understanding the relationship between CNV and cancer remains a major challenge. The unprecedented abundance of CNV, gene, and disease label data provides us with an opportunity to design a new machine learning framework to predict potential disease-related CNVs. In this paper, we developed a novel machine learning approach, namely, IHI-BMLLR (Integrating Heterogeneous Information sources with Biweight Mid-correlation and L1-regularized Logistic Regression under stability selection), to predict the CNV-disease path associations by using a data set containing CNV, disease state labels, and gene data. CNVs, genes, and diseases are connected through edges and then constitute a biological association network. To construct a biological network, we first used a self-adaptive biweight mid-correlation (BM) formula to calculate correlation coefficients between CNVs and genes. Then, we used logistic regression with L1 penalty (LLR) function to detect genes related to disease. We added stability selection strategy, which can effectively reduce false positives, when using self-adaptive BM and LLR. Finally, a weighted path search algorithm was applied to find top *D* path associations and important CNVs. The experimental results on both simulation and prostate cancer data show that IHI-BMLLR is significantly better than two state-of-the-art CNV detection methods (i.e., CCRET and DPtest) under false-positive control. Furthermore, we applied IHI-BMLLR to prostate cancer data and found significant path associations. Three new cancer-related genes were discovered in the paths, and these genes need to be verified by biological research in the future.

## Introduction

Copy number variations (CNVs) contribute to a substantial fraction of human genetic variation and are increasingly involved in disease associations and genome evolution (Lupski, 2015). Many evidences reveal the causal relationship between CNVs and many human disease phenotypes, including scores of known genomic diseases and hundreds of complex disease traits (Usher and McCarroll, 2015; Zarrei et al., 2015; Lauer and Gresham, 2019). One of the essential issues in CNV research is to understand how CNVs affect the occurrence of diseases (La Cognata et al., 2017; Gentile et al., 2021).

With the increase in the number of verified CNV–disease associations, several databases have been published, such as DGV (MacDonald et al., 2014), DGVa (Lappalainen et al., 2012), dbVar (Church et al., 2010), CNVD (Qiu et al., 2012), and DECIPHER (Firth et al., 2009). However, known CNV–disease associations include only a small fraction of CNVs and diseases. Calculation models and methods have been developed to predict the potential CNV–disease associations, which can be used as candidates for biological experimental verifications. Calculation models and methods would greatly reduce the experiment cost and save time in finding new CNV–disease associations.

The calculation methods can mainly be categorized into statistical classification-based and machine learning-based methods. People use statistical classification methods to develop innovative solutions to identify disease-related CNVs. For example, Shao et al. (2019) found that CNV is highly correlated with differential gene expression by counting the correlation between CNV and gene expression in a large number of cell lines and disease samples. Pan et al. (2019) proposed a calculation method that integrates Monte Carlo feature selection and incremental feature selection to identify discriminative core CNVs in different breast cancer subtypes. Xiong et al. (2012) proposed a single statistical framework, GSAA, which simultaneously measures genetic variation and gene expression variation across the entire genome to identify gene sets that are differentially expressed and thus can be used as markers related to studied traits. Reid et al. (2019) performed a genome-wide association study of common (>1%) CNV regions (CNVRs) with EOC (epithelial ovarian cancer) and HGSOC (high-grade serous) risk, and performed *in silico* analyses of tumor-gene expression. Barnes et al. (2008) presented a statistical framework for case–control CNV association study, which uses likelihood ratio to test differences between case and control samples.

Recently, many researchers are committed to using machine learning-based methods to study the complex mechanisms between CNVs and diseases. Lu et al. (2011) used Pearson correlation coefficient and pathway analysis to perform concurrent genome-wide analyses of CNVs and gene expression to identify gene reproducibly associated with tumorigenesis and survival in non-smoking female lung adenocarcinoma. Xu et al. (2018) proposed a support vector machine (SVM) classifier based on arm-level CNV data to detect early colorectal cancer. Onsongo et al. (2016) applied random forest to next-generation sequencing to detect CNVs. CCRET (Tzeng et al., 2015) collectively modeled the effects of multiple CNV features by measuring variants on a multi-categorical scale to find disease-related CNVs. Kim et al. (2012) introduced CNVRuler for CNV-association studies. CNVRuler supports chi-squared and Fisher’s exact tests in addition to logistic and linear regression analyses using defined CNVRs and clinical information. DPtest (Cheng et al., 2018) used a double penalty model to capture CNVs’ association with both the intensities and the disease traits. Zhang et al. (2019) proposed an ensemble learning framework ensembleCNV. ensembleCNV combines multiple individual CNVs with complementary strengths into CNVRs by using heuristic algorithm and then performs disease-related analysis on each CNVR through a global likelihood model.

Overall, the results of existing machine learning-based methods show that integrating diverse CNV-related information, disease-related information, and machine learning methods can boost the prediction accuracy of the CNV–disease association. However, most existing methods are limited to CNV and disease data. The prediction results contain many false-positive results (i.e., CNV not related to disease is identified as disease-related CNV) due to lack of consideration of the role of gene in CNV–disease association mechanism. In addition, most methods calculate the CNV/disease similarities only on those that have at least one known CNV–disease association.

**FIGURE 1:**
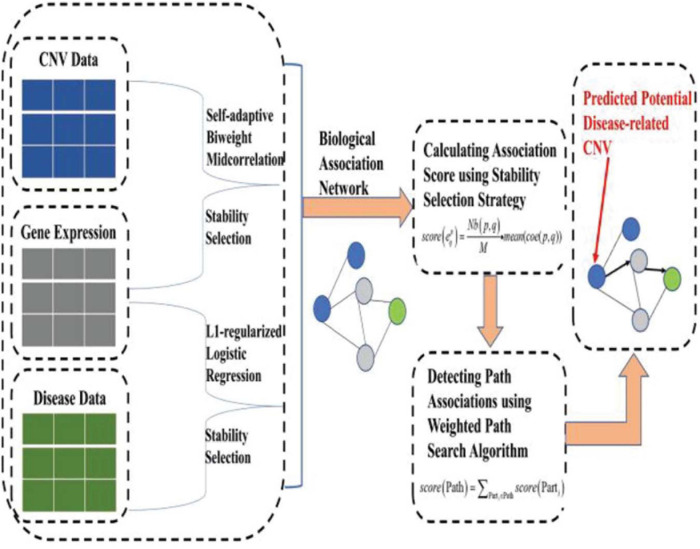
The structure of the IHI-BMLLR.

To address the aforementioned issues (or limitations), based on our previous work (Yuan and Huang, 2019), we put forward a novel machine learning approach, namely, IHI-BMLLR (Integrating Heterogeneous Information sources with Biweight Mid-correlation and L1-regularized Logistic Regression under stability selection), to predict the CNV–disease path associations by using a data set containing CNV, disease state labels, and gene data. IHI-BMLLR uses the three kinds of data to discover paths from CNV to disease. It should be noted that path means an association from a CNV to a gene and from the gene to disease. There is a biological association network where nodes represent CNVs, diseases, or genes and edges with scores representing the correlation between a pair of nodes. CNVs, genes, and diseases are connected through edges and then constitute a biological association network. To construct a biological network, we first used a self-adaptive biweight mid-correlation (BM) formula to calculate correlation coefficients between CNVs and genes. Although the Pearson correlation coefficient (PCC) is a widely used correlation coefficient calculation method, PCC is strongly affected while the BM remains practically the same as without the outliers (Langfelder and Horvath, 2012; Zheng et al., 2014; Yuan et al., 2015). Meanwhile, we used logistic regression with L1 penalty function (LLR) (Tibshirani et al., 2005) to detect genes related to disease. We added stability selection (SS) strategy (Meinshausen and Bühlmann, 2010; Yuan et al., 2018), which can effectively reduce false positives, when using self-adaptive BM and LLR. Finally, a weighted path search algorithm was applied to find top *D* path associations and important CNVs. Figure 1 illustrates the structure of the IHI-BMLLR method.

Compared with the traditional CNV–disease association analysis methods, our proposed approach has the following advantages. Firstly, compared with single CNV analysis, IHI-BMLLR can detect weighted associations, and consider all CNVs and genes simultaneously. Secondly, IHI-BMLLR uses three kinds of data (CNV, gene expression and disease state label data), which can use more information about CNV–disease mechanisms and provide insight into CNV–disease complex association mechanisms. Thirdly, the self-adaptive BM and weighted path search algorithm can help IHI-BMLLR accurately identify disease-related CNVs. Finally, because IHI-BMLLR does not require prior information, it is more suitable for large-scale data lacking complete prior information.

In the experiment section, we first compared the receiver operating characteristic (ROC) performance of IHI-BMLLR with two state-of-the-art CNV detection methods (CCRET and DPtest) using four kinds of simulation data. The experimental results show that IHI-BMLLR can significantly improve the detection performance of disease-related CNVs using gene data. From the results of the boxplots, we can see that the stability of IHI-BMLLR is better than CCRET and DPtest. Prostate adenocarcinoma (PRAD) is the most common cancer for males and the second death rate caused by cancer in men (Jemal et al., 2011). IHI-BMLLR was applied to PRAD data and obtained many CNV–disease path associations on the PRAD data from The Cancer Genome Atlas (TCGA) (Tomczak et al., 2015). IHI-BMLLR identified 212 significant paths, among which we analyzed top 10 path associations and calculated statistical significance of CNVs and genes in the paths. We used real and fake data test to calculate statistical significance of the top 10 CNV–disease path associations. The software and data of IHI-BMLLR are available at https://github.com/nathanyl/IHI-BMLLR.

## Materials and Methods

### Simulation Data and Prostate Cancer Data

In the “Results and Discussion” section, we compared IHI-BMLLR with CCRET and DPtest on simulation data and prostate cancer data. In this section, we will introduce simulation data set and prostate cancer data. Simulation data set contains four kinds of data sets with the same number of CNV–gene true associations, the same number of features, and different sample sizes (i.e., 1,000 CNVs, 100 genes, and 1 disease state). In simulation data set, the number of CNV–gene true associations is 100 CNVs–10 genes associations, the number of samples *N* ∈ {200, 500, 800, 1100}. The simulation data were generated as follows. Firstly, we generated 100 causal CNVs that are related to disease. The state label of the data is an equal number of diseases or normal states (i.e., the same number of 0s and 1s; 0 means normal state, 1 means disease state). Secondly, we used a three-layer fully connected neural network to generate 10 gene expression data. In the three-layer fully connected neural network, the input layer contains 100 nodes, the middle layer contains 10 nodes, and the output layer contains 1 node. The nodes in the input layer represent CNVs, the nodes in the middle layer represent genes, and the nodes in the output layer represent disease. Thirdly, we used the TensorFlow with the back propagation (BP) algorithm to train the three-layer fully connected neural network until the neural network can correctly predict more than 95% of disease state label nodes (Abadi et al., 2016). We used the values of the middle layer nodes as gene expression values, and the values in the input layer nodes were mapped to [−2, −1, 0, 1, 2]. Finally, we added 900 CNV values and 90 gene values to the sample. The CNV values were randomly selected from [−2, −1, 0, 1, 2], and the gene values were from Gaussian distribution. The 900 CNVs and 90 genes represent CNVs/genes that are not related to the diseases. Meanwhile, we also added noise data from Gaussian distribution *N*(0,1) to the data.

The PRAD data were downloaded from https://xenabrowser.net/ (Goldman et al., 2017). The CNV profile of PRAD was measured experimentally using genome microarray and the GISTIC2 method (Mermel et al., 2011; Izzi et al., 2020). The Illumina HiSeq 2000 RNA Sequencing platform was used to measure the gene expression profile (Fumagalli et al., 2014). The corresponding disease state label data were downloaded from TCGA Pan-Cancer Clinical Data Resource (TCGA-CDR) (Ge et al., 2016; Liu et al., 2018). We ran simulation and prostate cancer data experiments on a computer with Intel Xeon W-3175X CPU and 256G RAM.

### Methods

In this paragraph, we introduce the notations used in this article. We used boldface uppercase to represent matrices, boldface lowercase for vectors, and lowercase letters for scalars. We denote the CNV genotype data matrix by *X* ∈ *R*^*N*×*P*^, where *N* is the sample size and *P* is the CNV number, *x*_*j*_ denotes the *j*th column of CNV data matrix, ^*xi*^ denotes the *i*th row of CNV data matrix, and $x_{j}^{i}$ is the (*i*, *j*) element of CNV data matrix. Gene expression matrix is represented by *Y* ∈ *R*^*N*×*Q*^ with *N* sample size and *Q* gene traits, and disease state label matrix is represented by *Z* ∈ *R*^*N*×*K*^ with *K* diseases.

In the following paragraphs, we introduce the methods in the machine learning framework of finding the CNV–disease path associations. We also introduce how to discover CNVs affecting genes, identify genes affecting diseases, construct a biological network, and define a mathematical formula to calculate the scores of the path associations. Finally, we show how to use a weighted path search algorithm to discover top *D* path associations and important CNVs. Figure 1 illustrates the structure of IHI-BMLLR method.

### Discovering Paths in a Biological Association Network

We constructed a biological association network, the nodes in the network are used to represent CNV, genes, and diseases. We describe how to establish a connection between two nodes using self-adaptive BM coefficient and LLR under SS strategy. Self-adaptive BM and LLR are powerful techniques to find correlations between CNVs and genes or correlations between genes and diseases, and the SS method is used to effectively control the number of false-positive results.

Based on the self-adaptive BM coefficient, we computed correlation coefficients between CNVs and genes (Langfelder and Horvath, 2012):

(1) $$u_{i}=\frac{x_{i}-m\,e\,d\,\left(x\right)}{\alpha •m\,a\,d\,\left(x\right)}$$

The parameter *α* is often set to 9 empirically. However, this setting does not consider the characteristic of the data. In this paper, we set *α* to the data-driven parameter (*mad*(**x**)+*med*(**x**))/2. The range of self-adaptive BM values is from −1 to 1. If the correlation between a pair of elements is stronger, the absolute value of BM is larger.

Next, Equations (2) and (3) are used to detect associations between diseases and genes, the logistic loss function is applied to measure the gap between predicted disease state and true disease state (i.e., mark the disease state as 1 and mark the normal state as 0). Given a gene expression vector **y** as follows:

(2) $$p\left(\text{z}=1|y;\theta \right)=\sigma \left(\theta^{T}y\right)=\frac{1}{1+\exp\left(-\theta^{T}\,y\right)}$$

where θ ∈ ^*RQ*^ represents coefficient value vector of logistic regression model in Equation (2), and σ(⋅) represents the sigmoid function; thus, logistic regression formula with L1 penalty function can be defined as follows:

(3) $$\min_{\theta }\sum_{i=1}^{N}-\log p\,\left({\text{z}}^{\left(i\right)}|y^{\left(i\right)};\theta \right)+\lambda \,{||\theta ||}_{1}$$

In practice, our proposed method IHI-BMLLR is used to study a class of diseases and the disease state matrix is denoted by *Z* ∈ *R*^*N*×1^. The regularization parameter can affect the performance of model; the regularization parameter *λ* is determined by cross-validation technique. However, we tend to get many false-positive results when only using the cross-validation technique (Meinshausen and Bühlmann, 2010; Yuan et al., 2017). We combine the SS strategy when using self-adaptive BM and LLR algorithms. We will introduce the SS strategy later.

### Calculating Association Score Using SS Strategy

In this paper, IHI-BMLLR uses self-adaptive BM and LLR with SS strategy to find connections in a biological network. We summarized IHI-BMLLR under SS in Algorithm 1. Stability selection strategy uses the resampling technique. Firstly, half of the sample is randomly selected *M* times from the overall sample; for each randomly selected data, self-adaptive BM and LLR are applied to the corresponding selected data set (i.e., self-adaptive BM is applied to data set containing CNV and gene expression data, and LLR is applied to data set containing gene expression and disease state data). Secondly, in *M* times experiments, if the number of non-zero absolute value of coefficient between CNV and gene or gene and disease is greater than or equal to *M*⋅ϕ times, then the CNV or gene will be retained. ϕ is a predefined parameter used to effectively control the number of false-positive results. People have done a lot of in-depth research on the choice of *M* and ϕ values. Meinshausen’s research show that when *M* is greater than or equal to 100 times, it is sufficient to control false positives (Meinshausen and Bühlmann, 2010). In practical application, researchers often set ϕ in the range from 0.5 to 1. The larger the value of ϕ, the better the false-positive control at the cost of a reduced true-positive rate. This parameter ϕ is a hyperparameter. In this article, we choose 0.7 and 0.8. When detecting the potential associations between CNVs and the *q*th gene, the mathematical formula between the number of false-positive results and ϕ is defined as follows.

(4) $$E\,\left(V_{q}\right)\leq \frac{1}{2\,\phi -1}\,\frac{c^{2}}{P}$$

**Algorithm 1:** IHI-BMLLR under stability selection.

	**Input:** **X:** CNV genotype data, **Y:** gene expression matrix, **Z:** disease state matrix, O: selected CNVs by screening, ϕ: threshold parameter for stability selection strategy (0.5≤ϕ≤1), and *M*: total number of random samples
	**Output:** *I*: selected edges with scores
1.	*Π_*l*_ = 0,*l* ∈ *O**
2.	Selecting *N*/2 samples from *N* samples using random sampling without replacement
3.	Given *N*/2 subsamples, IHI-BMLLR, findλusing cross-validation, denoted by {λ*}
4.	*o*_*l*_ = 0, ∀*l* ∈ *o*
5.	**For** *t* = 1 to *M* **do**
6.	Selecting *N*/2 samples from *N* samples using random sampling without replacement
7.	Given *N*/2 subsamples, solve IHI-BMLLR with {λ*}
8.	*o*_*l*_ = *o*_*l*_ + 1 for all selected *l*
9.	Given the remaining λsubsamples, solve IHI-BMLLR with {λ*}
10.	*o*_*l*_ = *o*_*l*_ + 1 for all selected *l*
11.	$\Pi_{l}\leftarrow \frac{o_{l}}{2\,T},\forall l\in o$
12.	**I* = (*l*, Π_*l*_) : Π_*l*_ ≥ ϕ*

where *E*(*V*_*q*_) represents the expected value of false-positive CNVs associated with the *q*th gene, and parameter *c* represents the number of non-zero associations found by the IHI-BMLLR method. From Equation (4), we can see that the upper limit of false-positive results is inversely proportional to the parameter ϕ. When we apply IHI-BMLLR to detect the association between genes and diseases, the same situation exists for the relationship between false positives and parameter ϕ.

After self-adaptive BM and LLR combined with the SS method is used in the data set, and after obtaining the result, we can calculate the significance scores of the connections in the biological association network. When detecting the relationship between the *q*th gene trait and the *p*th CNV, the significance score of the association can be defined as follows:

(5) $$s\,c\,o\,r\,e\,\left(e_{q}^{p}\right)=\frac{N\,b\,\left(p,q\right)}{M}•m\,e\,a\,n\,\left(c\,o\,e\,\left(p,q\right)\right)$$

where $e_{q}^{p}$ represents the association between the *p*th CNV and *q*th gene. *Nb*(*p*,*q*) represents the number of data sets in which $e_{q}^{p}$ is accurately found. Obviously, the value of *Nb*(*p*,*q*)/*M* is in the range 0–1. *mean*(*coe*(*p*,*q*)) represents the average value of the accurately identified correlation coefficients. The larger the value of score ($e_{q}^{p}$), the greater the correlation between the *p*th CNV and the *q*th gene in the biological network.

Based on the association score between two nodes in the biological network, we can calculate the score of path that contains a CNV, a gene, and a disease. The association path composed of important connections can be regarded as a significant biological association path. In order to find important paths efficiently, we use a weighted path search algorithm; the details of the algorithm will be described in the next section.

### Detecting Path Associations Using Weighted Path Search Algorithm

There are a large number of associated paths in a complex heterogeneous biological network. In this paper, a path represents a continuous biological path in which a CNV is connected to a disease through a gene. In order to accurately find important path associations, a weighted path search algorithm was used to calculate the significance scores of paths and find important biological paths (i.e., high-score association paths) (Yuan et al., 2018). In the biological network, significant paths tend to have larger scores.

In a biological association network, a weighted path search algorithm can be defined as follows. Firstly, in the biological network, we choose genes that are simultaneously associated with CNV and disease. We can find all existing association paths by selecting these genes. Secondly, we can obtain the score of paths by summing the weighted scores [Equation (5)] of each part of the path. Finally, we sort all paths in descending order of scores and select the top *D* high-score path associations. The weighted path score formula can be defined as follows:

(6) $$s\,c\,o\,r\,e\,\left(\text{Path}\right)=\sum_{{\text{Part}}_{i}\in \text{Path}}s\,c\,o\,r\,e\,\left({\text{Part}}_{i}\right)$$

### Criteria for Evaluating Method Performance

**FIGURE 2:**
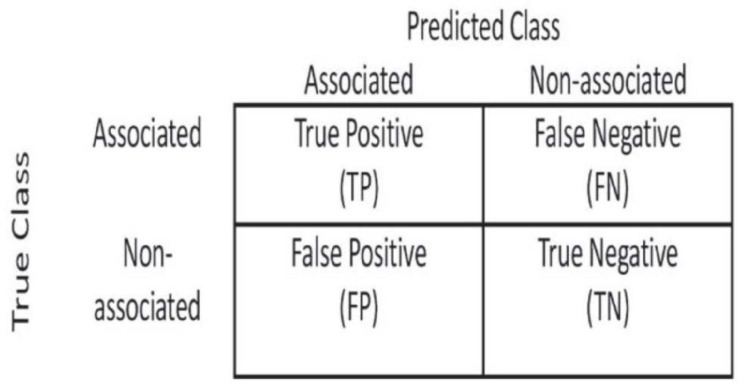
Confusion matrix used to calculate TPR and FPR.

In order to evaluate the performance of methods fairly, we used the ROC curve to observe the performance of methods and compared the performance of methods using the area under receiver operating characteristic (AUROC). We calculated TPR and FPR based on the confusion matrix (Figure 2).

### Disease-Related CNV True Labels Test

In the disease-related CNV true labels test, FaST-LMM-EWASher (Zou et al., 2014), which is a conventional linear regression method, was used to predefine disease-related CNVs. The predefined CNVs are treated as true labels. Then, we compared performance of IHI-BMLLR, CCRET, and DPtest in accurately identifying the true labels. An excellent disease-related CNV detection method should report as many true labels as possible in the resulting CNVs.

### Real Data and Fake Data Test

In the designed real data and fake data test, three methods (i.e., IHI-BMLLR, CCRET, and DPtest) were applied to real CNV–disease data and obtained a set of “real” results; then, these three CNV detection methods were applied to “fake” data that exchange samples between two category conditions and obtained a set of “fake” results. Compared with the expected biologically significant “real” results, the “fake” results have no biological significance. An excellent CNV detection method should find as many CNVs as possible in the “real” results, while reporting as few CNVs as possible in the “fake” results. In addition, when methods find the same amount of CNVs on the “fake” data set, the method that detects more CNVs on the “real” data set has better performance.

### The Statistical Significance Calculation Method of Paths

In order to measure the statistical significance of the 10 paths, we used a method of calculating statistical significance by comparing the original path score and the random path score. In bioinformatics research, this is a widely used statistical significance calculation method (Mootha et al., 2003; Liberzon et al., 2015; Wei et al., 2016; Yuan et al., 2016). Firstly, we randomly rearranged CNV samples, gene samples, and disease labels, and then calculated the scores of the paths. Secondly, we repeated the previous steps 5,000 times, calculated the score of the path in each randomly generated sample, and then constructed a histogram of the scores. Thirdly, we calculated the *p*-value of the path by calculating the proportion of the path score in 5,000 random data that is less than the path score in the original data.

The null hypothesis in this paper is that path scores from random data are randomly distributed scores. The alternative hypothesis is that the path score is related to the structure of the sample data. Assuming that the *p*-value of score is 0.001, this means that there are random path scores less than the original path score under null hypothesis.

## Results and Discussion

Our study firstly compared performance of IHI-BMLLR with two state-of-the-art methods (i.e., CCRET and DPtest) using AUC in the simulation data. The results show that IHI-BMLLR performs clearly better than other methods. Then, we compared the stability performance of these three methods using boxplots. The results show that the stability of IHI-BMLLR is better than the other two methods. IHI-BMLLR also achieved a better performance in test for real and fake data. In order to find the path-related information in cancer, we applied IHI-BMLLR to PRAD data from TCGA. The results contained 212 path associations. We used disease-related CNV true labels test to calculate the statistical significance of the top 10 high-scoring path associations, and further analyzed the statistical significance of CNVs and genes in the top 10 paths.

### Comparison of Methods on Simulation Data

**FIGURE 3:**
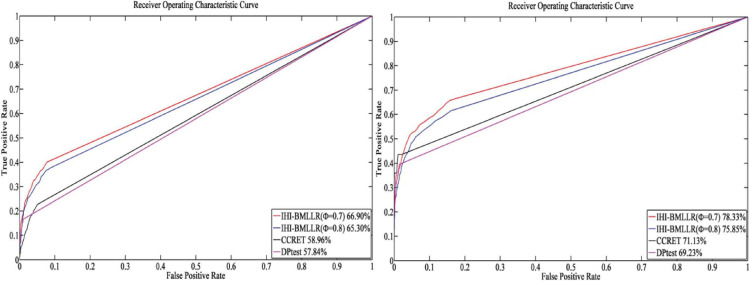
ROCs of IHI-BMLLR with CCRET and DPtest: *N* = 200 (left), *N* = 500 (right). For IHI-BMLLR, we show the results with two settings for ϕ 0.7 and 0.8.

**FIGURE 4:**
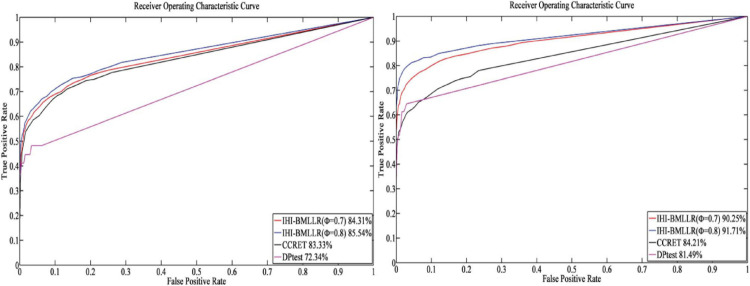
ROCs of IHI-BMLLR with CCRET, and DPtest: *N* = 800 (left), *N* = 1,100 (right). For IHI-BMLLR, we show the results with two settings for ϕ 0.7 and 0.8.

For the parameters in method IHI-BMLLR, we set ϕ 0.7 and 0.8, *M* = 100, and *D* = 2,000. Figures 3, 4 show the ROCs obtained by IHI-BMLLR with two parameter settings ϕ = {0.7,0.8}. Panels show the results for different sample sizes (*N* ∈ {200, 500, 800, 1100}). Supplementary Figures 1, 2 show the corresponding AUC values of Figures 3, 4, respectively.

**FIGURE 5:**
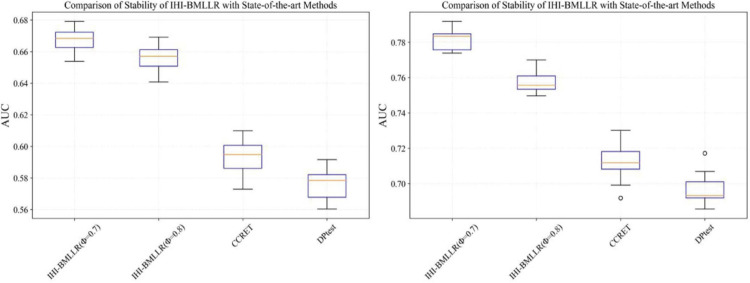
The boxplots of the AUCs for IHI-BMLLR, CCRET, and DPtest with different sample sizes. *N* = 200 (left), *N* = 500 (right).

**FIGURE 6:**
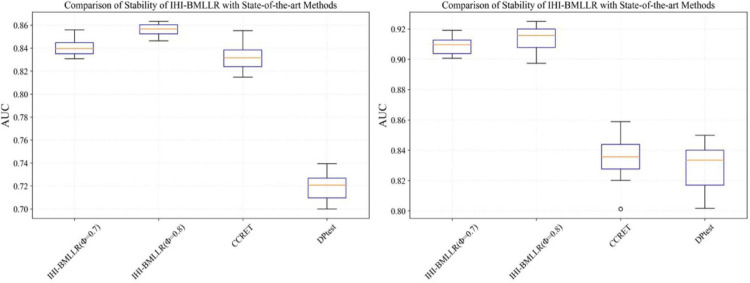
The boxplots of the AUCs for IHI-BMLLR, CCRET, and DPtest with different sample sizes. *N* = 800 (left), *N* = 1,100 (right).

It can be seen that in the ROC and AUC results, the IHI-BMLLR achieved higher AUC values regardless under both values of the ϕ parameter. The results suggest that when the pathogenesis mechanism of the disease is complex, for example, when CNVs affect the disease through a complex transmission mechanism, two biological factors association (i.e., CNVs and disease or CNVs and genes) analysis may not accurately find the causal CNVs that affect the disease. Each kind of simulation data set was randomly generated 100 times. We calculated the AUC value of each method 100 times and then generated the corresponding boxplot. Figures 5, 6 show the boxplots. As shown in Figures 5, 6, the stability of IHI-BMLLR with different parameters is much better than the other two methods, and the experimental results of our proposed method do not contain outliers.

### Comparison of Methods on PRAD Data

PRAD is the most common cancer for males and the second death rate caused by cancer in men. Research shows that CNV makes an important contribution to the proliferation of PRAD malignant cells (Laitinen et al., 2016). CNV–disease path associations (i.e., the association between CNVs and diseases through genes) can provide biological information for in-depth understanding of the complex mechanisms of cancer. Therefore, IHI-BMLLR, CCRET, and DPtest were applied to the PRAD data from TCGA. PRAD data contain CNV and gene expression profiles of 490 samples. The data set includes 24,776 CNVs and 20,530 DNA probe expression values from the same sample, which includes known and predicted genes. Binary labels (i.e., 1 denotes disease state and 0 denotes normal state) were used to indicate the sample state.

**FIGURE 7:**
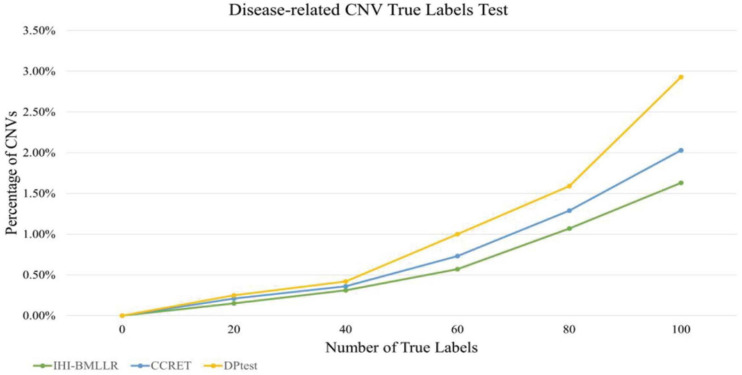
Comparison of IHI-BMLLR, CCRET, and DPtest in the PRAD data CNV true labels test experiment.

The main limitation of using real data sets to test disease-related CNV analysis methods is the lack of experimentally verified CNV data. The lack of verification data makes it difficult to effectively evaluate the performance of a method. In order to effectively compare the performance of various methods, true labels test was applied to three methods. Firstly, FaST-LMM-EWASher was used to identify the PRAD-related CNVs; then, these CNVs are defined as true labels. Secondly, we selected the top 100 CNVs from the PRAD-related CNVs. Because these 100 CNVs are closely related to the development of PRAD, the method should discover as many CNVs as possible. Finally, we compared the performance of methods in discovering these true labels. The experimental results are shown in Figure 7. As shown in Figure 7, the method IHI-BMLLR performs significantly better than the other two methods. Supplementary Table 1 contains the detail information of results.

**FIGURE 8:**
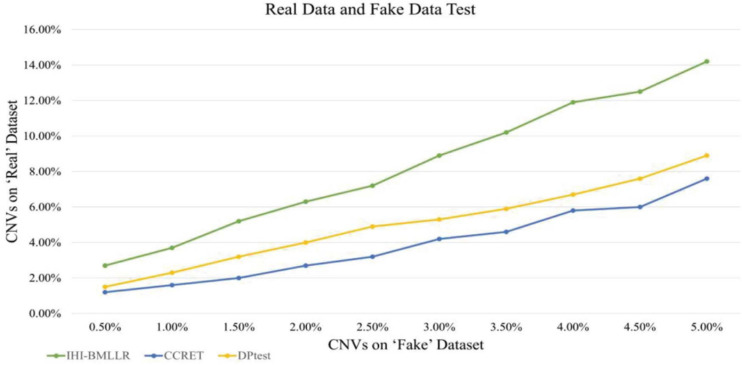
Comparison of IHI-BMLLR, CCRET, and DPtest in the PRAD real data and fake data test.

We also used real data and fake data test to evaluate and compare the performance of three disease-related CNV detection methods. When the ground truth is unknown, the test method is widely used in bioinformatics research (Zhang et al., 2008; Cui et al., 2015, 2016; Liu et al., 2017). As shown in Figure 8, IHI-BMLLR outperforms the two state-of-art methods. For IHI-BMLLR, when 3.7% of CNVs were found on the “real” data set, at the same time, 1% of CNVs were found on the “fake” data set. Supplementary Table 2 contains the numerical information of Figure 8.

### Finding Path Associations From PRAD Data

We used IHI-BMLLR with 10-fold cross-validation to find the path associations. We set ϕ = 0.7and *M* = 100. The parameter ϕ is set to 0.7 to ensure that as many biologically meaningful paths as possible are included in the result. A path contains a CNV, a gene, and the disease. We found 212 paths in the PRAD data results. It should be noticed that the maximum path score is 2. We studied and analyzed the top 10 high-score paths, which contain three prostate oncogenes *PCGEM1* (Srikantan et al., 2000), *ERG* (Adamo and Ladomery, 2016), and *MXI1* (Huang et al., 2018). The paths and corresponding statistical analysis value are shown in Table 1.

**TABLE 1:** Top 10 path associations found by IHI-BMLLR in the PRAD data related to *PCGEM1*, *ERG*, and *MXI1*.

**Chromstart**	**Chromend**	**Gene**	**Path score**	**Path *P*-value**
*chr*4*:*132639974	132640994	*PCGEM1*	1.96	0.011
*chr*2*:*162563851	162564769	*PCGEM1*	1.95	0.01
*chr*5*:*89153624	89153750	*PCGEM1*	1.94	0.009
*chr*11*:*115579831	115582002	*PCGEM1*	1.93	0.012
*chr*6*:*121675165	121694284	*ERG*	1.93	0.008
*chr*2*:*141069793	141080710	*ERG*	1.92	0.017
*chr*5*:*100264445	100264533	*ERG*	1.91	0.009
*chr*16*:*56055045	56056797	*MXI1*	1.85	0.033
*chr*2*:*136738245	136739236	*MXI1*	1.84	0.015
*chr*6*:*75204004	75207834	*MXI1*	1.84	0.021

### Significant Analysis of CNVs and Gene in Independent Data

In order to verify whether CNV has a specific function, we used independent data GSE79402 to calculate the Student’s *t*-test *P*-values and *T*-scores. As shown in Table 2, the *P*-values of 10 CNVs are all less than 1E–10. The *P*-values indicate that we can reject the null hypothesis and consider that the biological functions of these 10 CNVs are significantly different under normal and disease states.

**TABLE 2:** The Student’s *t*-test *P*-values and *T*-scores of 10 CNVs.

**Chrom**	**Chromstart**	**Chromend**	***P*-value**	***T*-score (case–control)**
4	132639974	132640994	6.12e-16	−12.503
2	162563851	162564769	3.24e-22	−11.004
5	89153624	89153750	8.12e-18	−10.314
11	115579831	115582002	7.12e-29	−6.741
6	121675165	121694284	4.12e-24	−16.147
2	141069793	141080710	8.13e-25	−13.218
5	100264445	100264533	5.38e-17	−17.194
16	56055045	56056797	6.12e-24	−12.933
2	136738245	136739236	5.32e-32	−7.572
6	75204004	75207834	6.14e-14	−14.372

In order to verify whether three oncogenes have different functions between prostate cancer cases and controls, we used Student’s *t*-test and Wilcoxon rank sum test to calculate the statistical significance of genes from independent data GSE60329. Table 3 contains the results of Wilcoxon rank sum test and Student’s *t*-test. In the result of Student’s *t*-test, the *P*-values of three oncogenes are all less than 1E–04. Meanwhile, in the result of Wilcoxon rank sum test, the *P*-values of three genes are all less than 1E–04. These two test results indicate that the three genes are significantly differentially expressed between prostate cancer cases and controls.

**TABLE 3:** The Student’s *t*-test *P*-values and Wilcoxon rank sum test for 10 CNVs.

**Method value**	***PCGEM1***	***ERG***	***MXI1***
Student’s *t*-test *P*-value	3.54e-07	1.35e-09	5.37e-05
Student’s *t*-test *T*-score (case–control)	15.3214	17.1090	9.3421
Wilcoxon rank sum test *P*-value	3.25e-08	2.34e-08	1.93e-04
Wilcoxon rank sum test *H*-value	1	1	1

To compare the ability of methods to find differentially expressed genes. Firstly, we used a widely used genome-wide differential expression analysis method edgeR (Laitinen et al., 2016) to find differentially expressed genes. Then, we applied the methods (IHI-BMLLR/CCRET/DPtest) to the data and calculated the number of differentially expressed genes found by each method. edgeR identified 100 differentially expressed genes; IHI-BMLLR, CCRET, and DPtest found 63 genes, 45 genes, and 27 genes, respectively.

## Discussion

We identified four paths that contain *PCGEM1*. *PCGEM1* produces a long non-coding RNA that is overexpressed in prostate cancer and may act as a marker for tumor progression (Safran et al., 2010; Orii and Ganapathiraju, 2012). Further biological research is needed to confirm the path associations found by method IHI-BMLLR. In other path associations not discussed in the paper, we detected three genes *MAPK13*, *MCM4*, and *CCNB2* that are not related to PRAD. These genes are reported to be related to bladder cancer (Zekri et al., 2015; Gao et al., 2018; Zhang et al., 2018). It is necessary to study the relationship between these genes and PRAD in the future.

The real biological regulation mechanism in the human body is much more complicated than what we assumed. For example, the relationship between genes has received extensive attention in disease research. In this article, IHI-BMLLR is dedicated to discovering paths from CNV to gene and from the gene to disease. In the future, we will study and try to propose a method for studying gene–gene associations and optimal association numbers in CNV–disease research.

Finally, the real biological regulation mechanism in the human body is much more complicated than what we assumed. lncRNA and miRNA often work together with CNV, and our method does not consider lncRNA and miRNA. In the future, we will study how to design a machine learning framework that simultaneously considers both lncRNA and miRNA.

## Conclusion

In this article, we designed a novel disease-related CNV detection method IHI-BMLLR, which uses CNV, gene, and disease data to find path associations. The method consists of two parts. The first part of the method is the association search method. It contains adaptive BM correlation coefficient formula and LLR. The second part contains SS strategy and weighted search path algorithm. These two methods were used to control false positives and identify paths, respectively. The result of simulation data experiment proves that IHI-BMLLR is significantly better than two state-of-the-art methods CCRET and DPtest. The result of the boxplots indicates that the stability of IHI-BMLLR outperforms the two methods. In the results of the PRAD data experiment, IHI-BMLLR identified 212 important paths. Disease-related CNV true labels test and real data and fake data test were used to calculate the statistical significance of the top 10 high-score path associations. We also discovered three potential PRAD-related genes.

## Data Availability Statement

The datasets presented in this study can be found in online repositories. The names of the repository/repositories and accession number(s) can be found in the article/ Supplementary Material.

## Author Contributions

LY conceived the method, conducted the experiments, and wrote the main manuscript text. LY and ZS designed the method. TS and JZ prepared the Figures 1–3. All authors reviewed the manuscript.

## Conflict of Interest

The authors declare that the research was conducted in the absence of any commercial or financial relationships that could be construed as a potential conflict of interest.
