# Supplementary material for: A Novel Computational Framework to Predict Disease-Related Copy Number Variations by Integrating Multiple Data Sources
Source: Front Genet. 2021 Jun 29;12:696956. doi: 10.3389/fgene.2021.696956 (PMC8276077; doi:10.3389/fgene.2021.696956)
Supplement: Supplementary file 1 [file Data_Sheet_1.doc]

### Integrated Analysis of CNV, Gene Expression and Disease State Data in Prostate Cancer

### ——Supplementary Materials

1. Figure S1


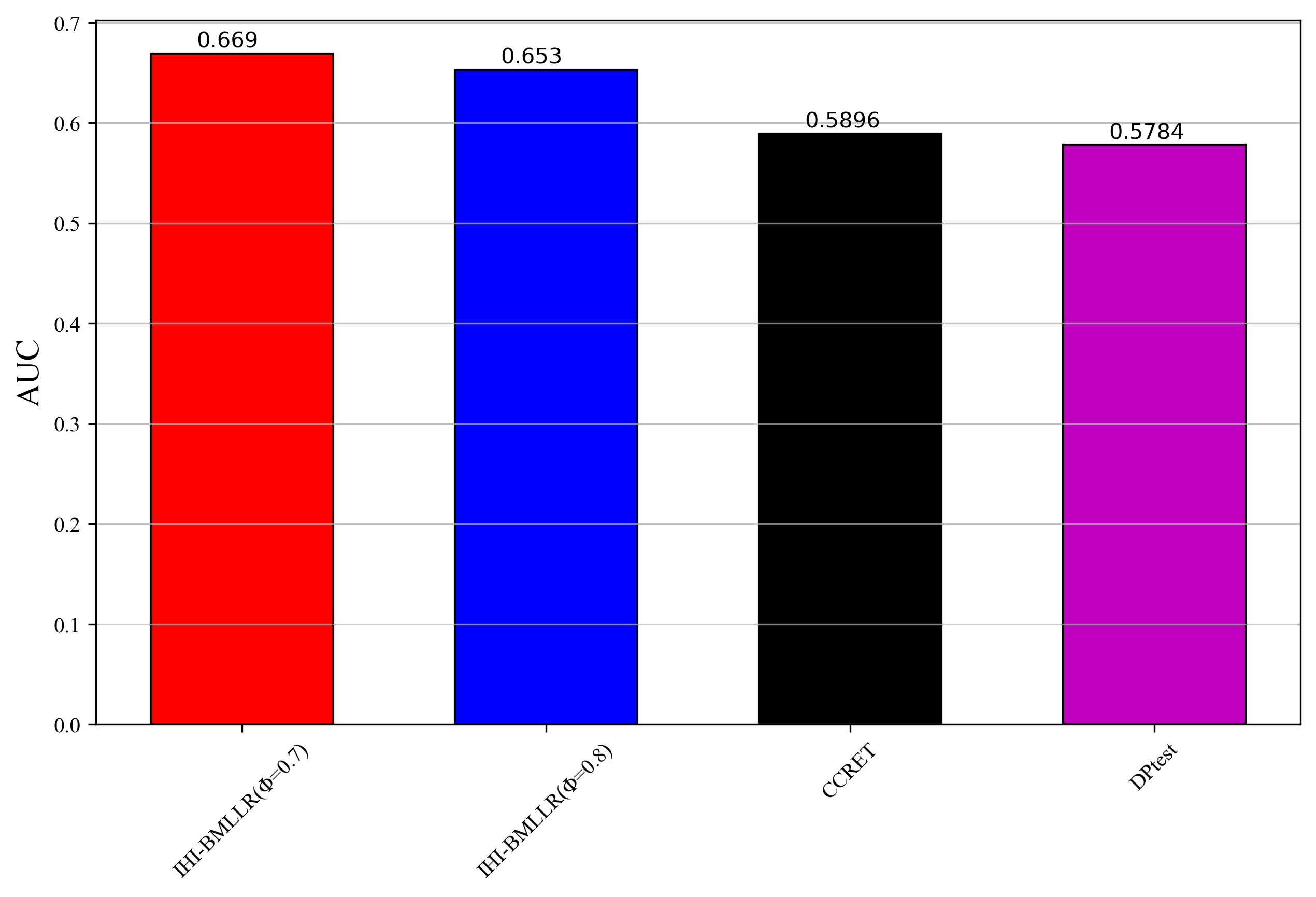


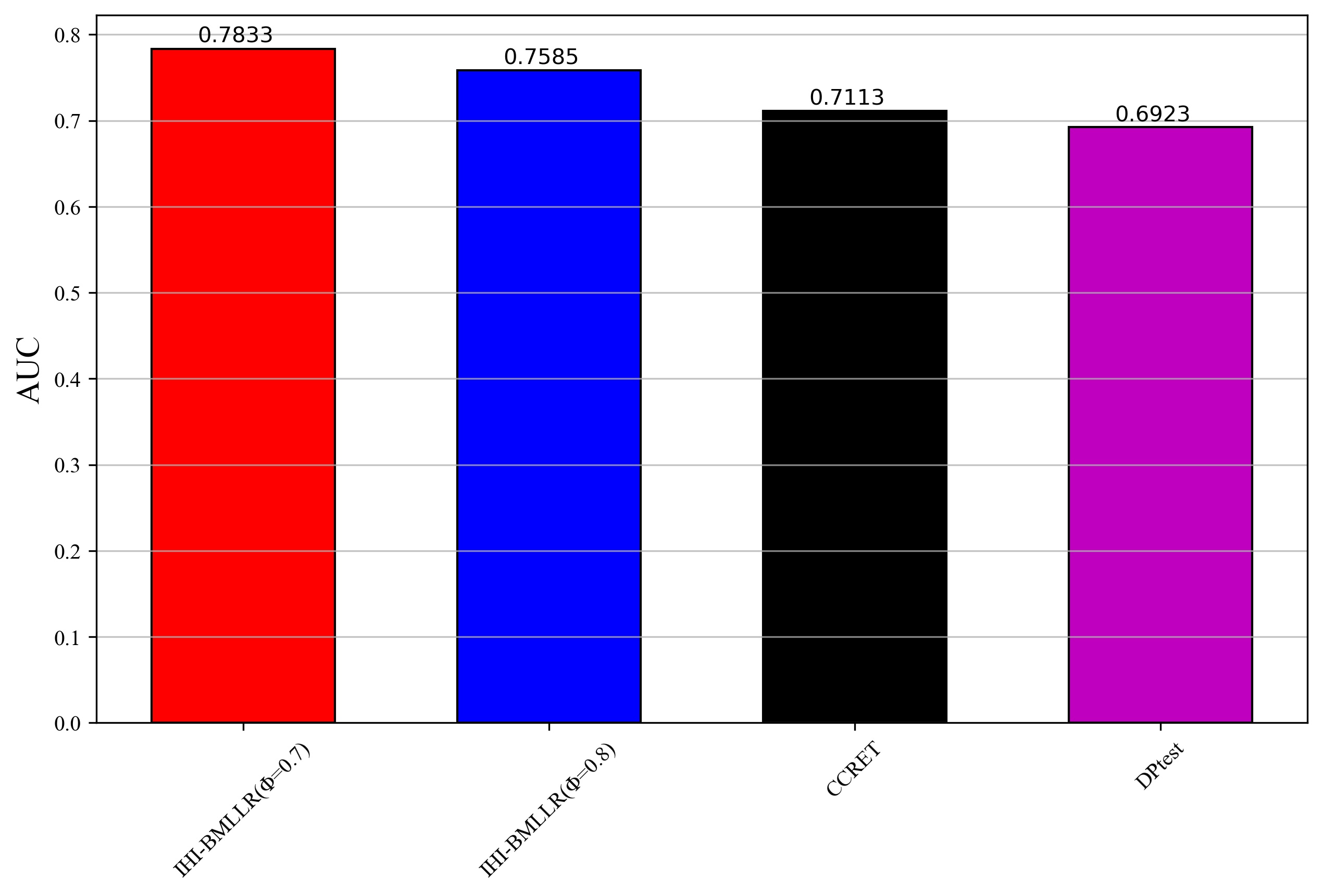


**Figure S1. AUC performances on simulation datasets (*N*=200 and *N*=500) for IHI-BMLLR with different and other comparative methods.** The AUC values in the figure are from Figure 3.

1. Figure S2


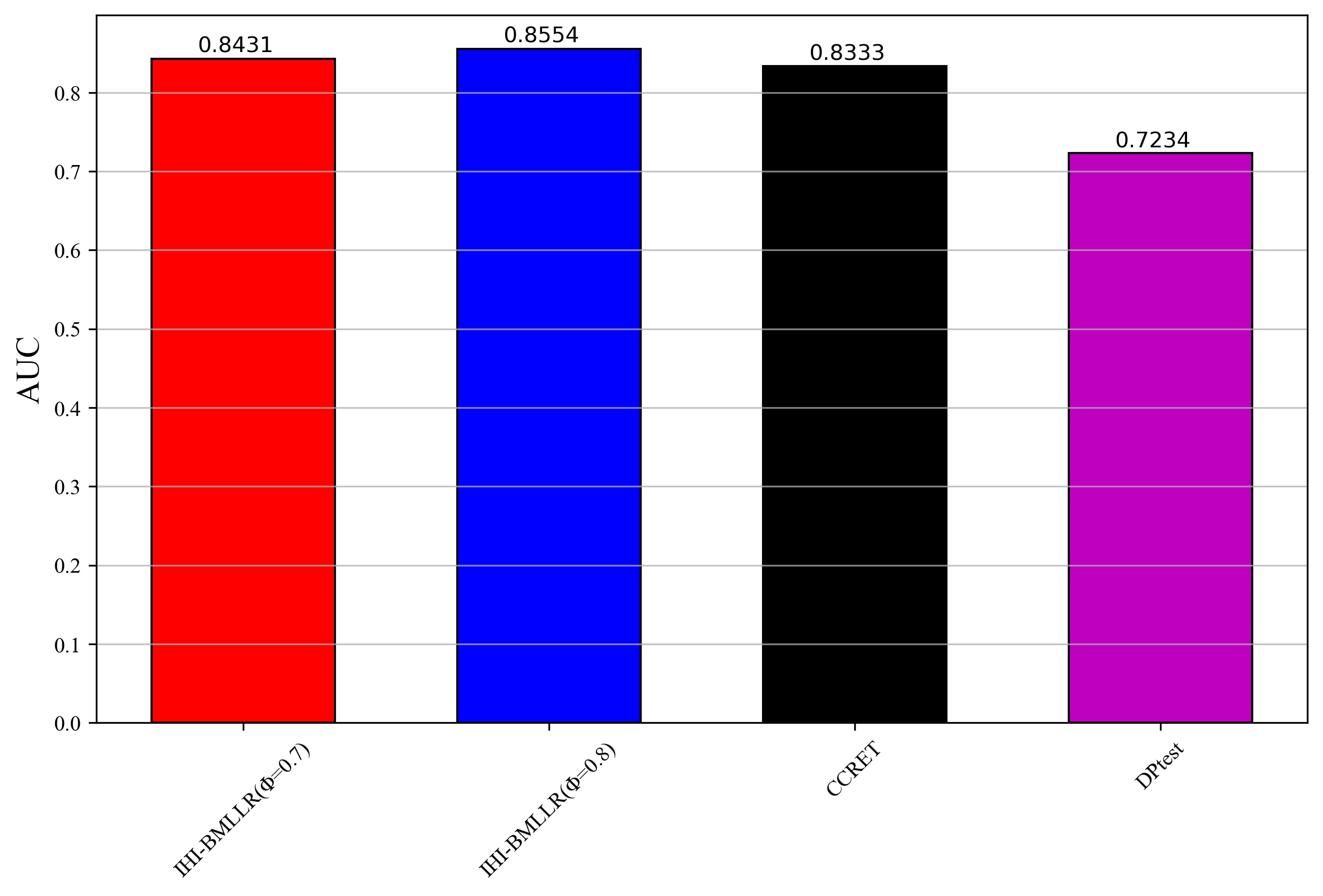


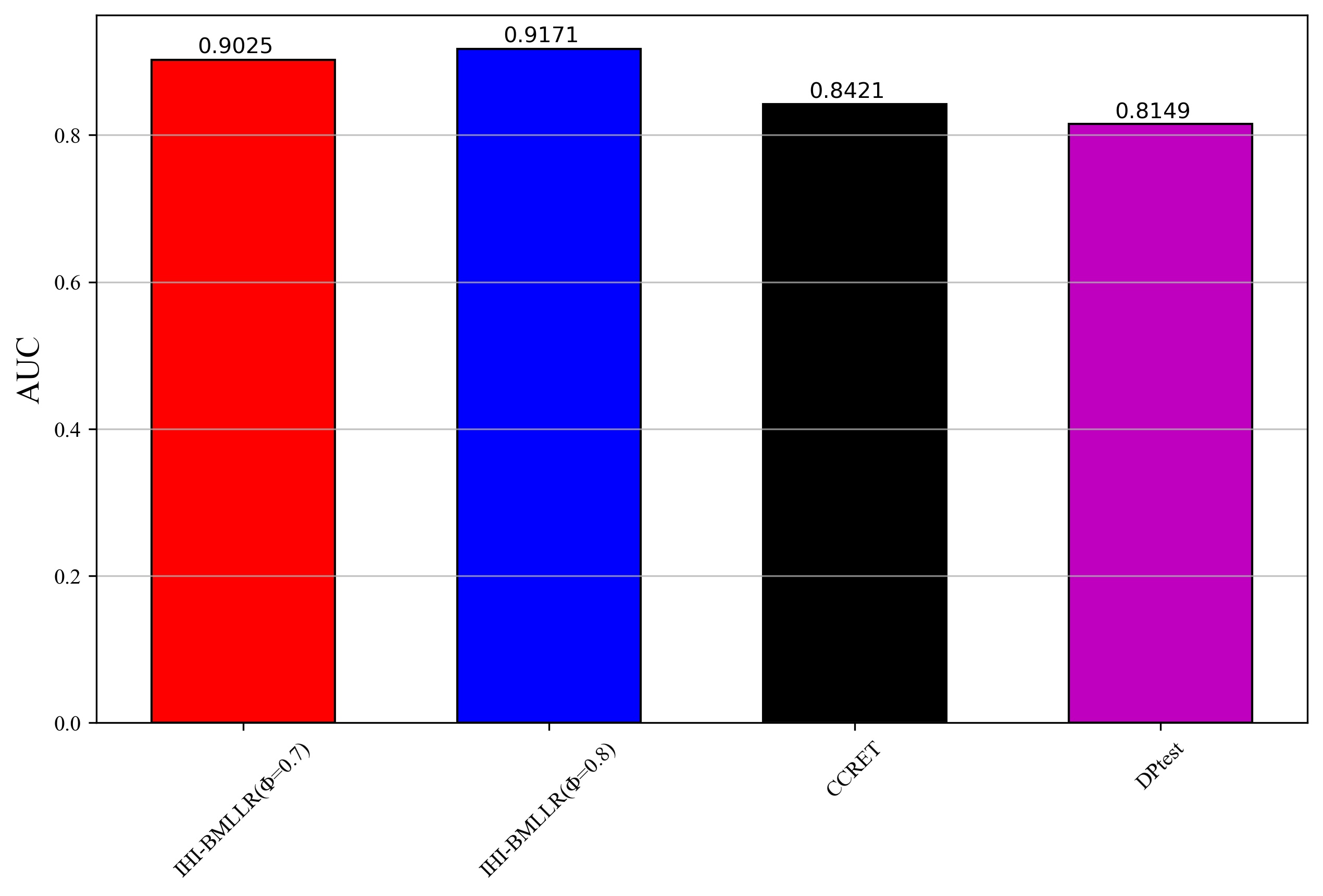


**Figure S2. AUC performances on simulation datasets (*N*=800 and *N*=1100) for IHI-BMLLR with different and other comparative methods.** The AUC values in the figure are from Figure 4.

1. 3. Table S1

| Number of true signatures | IHI-BMLLR | CCRET | DPtest |
| --- | --- | --- | --- |
| 0 | 0.00% | 0.00% | 0.00% |
| 20 | 0.15% | 0.21% | 0.25% |
| 40 | 0.31% | 0.36% | 0.42% |
| 60 | 0.57% | 0.73% | 1.00% |
| 80 | 1.07% | 1.29% | 1.59% |
| 100 | 1.63% | 2.03% | 2.47% |

**Table S1. Detail information of Figure 7.**

**4. Table S2**

| CNVs on "mock" dataset | IHI-BMLLR | CCRET | DPtest |
| --- | --- | --- | --- |
| 0.5% | 2.7% | 1.2% | 1.5% |
| 1.0% | 3.7% | 1.6% | 1.7% |
| 1.5% | 5.2% | 2.0% | 1.9% |
| 2.0% | 6.3% | 2.7% | 2.4% |
| 2.5% | 7.2% | 3.2% | 3.1% |
| 3.0% | 8.9% | 4.2% | 4.1% |
| 3.5% | 10.2% | 4.6% | 4.9% |
| 4.0% | 11.9% | 5.8% | 5.3% |
| 4.5% | 12.5% | 6.0% | 6.2% |
| 5.0% | 14.2% | 7.6% | 7.2% |

**Table S2. Detail information of Figure 8.**
